# Fulminant myocarditis induced by immune checkpoint inhibitor nivolumab: a case report and review of the literature

**DOI:** 10.1186/s13256-021-02934-y

**Published:** 2021-07-06

**Authors:** Feifei Wang, Yang Liu, Wei Xu, Changjing Zhang, Jianhong Lv, Shaolin Ma

**Affiliations:** Department of Intensive Care Unit, Shanghai East Hospital, Tongji University, Shanghai, China

**Keywords:** Nivolumab, Immune checkpoint inhibitor, Fulminant myocarditis, ECMO

## Abstract

**Background:**

Nivolumab, an anti-programmed cell death protein 1 antibody, is commonly used as an immune checkpoint inhibitor in various cancers. Various adverse events are associated with these therapies, including hepatitis, dermatitis, and myocarditis. Myocarditis is a relatively rare but potentially fatal immune-mediated adverse reaction.

**Case presentation:**

We report a case of colon cancer in a 56-year-old Chinese patient with lung and liver metastasis who developed fulminant myocarditis by nivolumab and survived with the support of extracorporeal membrane oxygenation. After six cycles (within 3 months) of nivolumab treatment, the patient developed chest tightness and was hospitalized. A diagnosis of fulminant myocarditis associated with immunotherapy was confirmed based on the clinical manifestations and laboratory examinations. He recovered well and was discharged on day 45 after management with extracorporeal membrane oxygenation, intravenous methylprednisolone, and immunoglobulin.

**Conclusions:**

This case illustrates a severe cardiovascular complication of immunotherapy, strongly suggesting the necessity of close monitoring for outpatient usage of nivolumab. Additionally, our experience provided an efficient management strategy of extracorporeal membrane oxygenation in terms of life-threatening conditions.

## Introduction

Immunotherapy with immune checkpoint inhibitors is increasingly recommended as a standard treatment for multiple malignancies. However, immune checkpoint inhibitors may cause severe immune-related adverse events, related to excessive immune activation [1]. Specifically, myocarditis is a low-probability complication but with extremely high mortality that deserves immediate recognition. The rate of myocarditis among fatalities associated with immunotherapy complications is 22%. It is reported that mortality of myocarditis is associated with immunotherapy [2]. So far, the main checkpoints that have been targeted by immunotherapy are cytotoxic T-lymphocyte activator-4 (CTLA-4), programmed-death-1 (PD-1), and its ligand, programmed death ligand 1 (PD-L1). Nivolumab, an anti-PD-1 monoclonal antibody, is an Ig (immunoglobulin) G4 monoclonal antibody that binds PD-1 on the surface of lymphocytes, allowing the immune system to recognize and destroy tumor cells which otherwise evade the immune response [3]. We present herein a patient who was successfully treated with extracorporeal membrane oxygenation (ECMO) for severe autoimmune fulminant myocarditis secondary to the immune checkpoint inhibitor nivolumab. This is the fifth case of fulminant myocarditis induced by immune checkpoint inhibitors supported with ECMO.

## Case report

A 56-year-old Chinese man was diagnosed with colonic carcinoma with liver and lung metastases (T2N0M1b, stage IV). His family history and social history were noncontributory. He underwent Hartmann operation in November 2016. The patient’s tumor tissue was classified as wild-type genotypes of dMMR, KRAS, NRAS, and BRAF. Then he was treated with 12 cycles of XELOX chemotherapy combined with cetuximab. The patient underwent hepatectomy at Mayo Clinic in the United States in December 2017 after treatment with a single 6 Gy dose of local liver radiation for six times. Afterward, he underwent folinic acid–fluorouracil–irinotecan (FOLFIRI) combined with panitumumab chemotherapy for seven cycles after the operation. Because chest and abdomen computed tomography (CT) scan revealed new lesions in the liver and lungs in November 2018, he received six cycles of immunotherapy with nivolumab (140 mg daily), an anti-PD-1 antibody, within 3 months. His myocardial enzymes and echocardiography were normal before nivolumab treatment. He presented progressive chest tightness and pain after 1.5 months of taking nivolumab. Laboratory tests after admission to hospital revealed elevated serum NT-proBNP (180.40 ng/L, normal < 125 ng/L), CK-MB (8.99 ng/mL, normal < 4.87 ng/mL) and troponin T (0.23 ng/mL, normal < 0.014 ng/mL). Transthoracic echocardiography showed a left ventricular ejection fraction (LVEF) of 50%. Electrocardiogram reported sinus bradycardia and inverted T wave in lead II, III, and aVF. Three days later, these laboratory tests showed further increased NT-proBNP (612.30 ng/L), CK-MB (12.33 ng/mL), and troponin T (0.49 ng/mL). Coronary artery CT angiography revealed no coronary artery lesions. The serological analysis of antiviral antibodies did not suggest myocarditis-associated viral infection. Considering the relation of onset time and the drug therapy, the most likely diagnosis was fulminant myocarditis secondary to nivolumab administration.

**Fig. 1:**
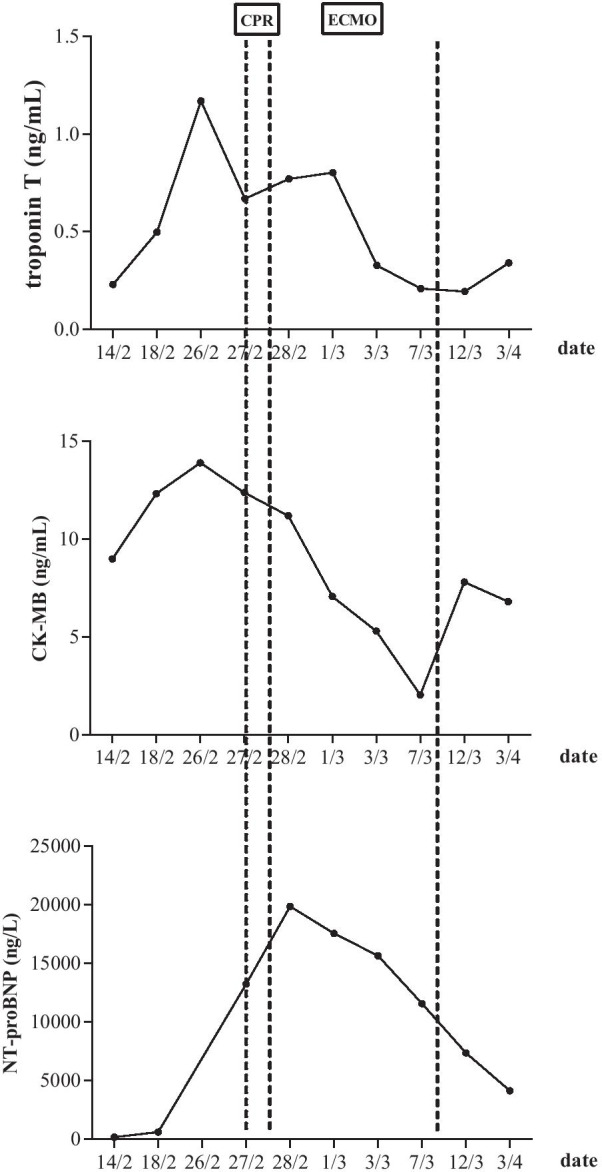
Timeline of NT-proBNP, CK-MB, and troponin T levels during hospitalization. The dotted line indicates the application time of CPR and ECMO. *ECMO* extracorporeal membrane oxygenation, *CPR* cardiopulmonary resuscitation

**Fig. 2:**
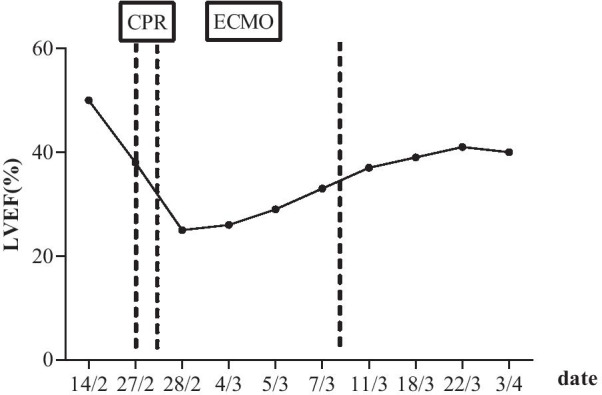
Timeline of LVEF during hospitalization. The dotted line indicates the application time of CPR and ECMO. *ECMO* extracorporeal membrane oxygenation, *LVEF* left ventricular ejection fraction, *CPR* cardiopulmonary resuscitation

The patient rapidly experienced cardiogenic shock and was transferred to intensive care unit after cardiopulmonary resuscitation and tracheal intubation. He revived within half an hour with blood pressure of 89/52 mmHg on the support of 8 µg/kg/minute dopamine and 0.12 µg/kg/minute epinephrine. NT-proBNP was higher than 15,000 ng/L (Fig. 1). Electrocardiogram showed a heart rate of 108 beats/minute with wide QRS and arrhythmia. Echocardiography revealed declined movement of the left ventricle multisegments and the right ventricle free wall, and LVEF was 25% (Fig. 2). The patient was unconscious and exhibited hypotension, tachycardia, high lactic acid, and anuria. Then, venoarterial extracorporeal membrane oxygenation (VA-ECMO) was used to support his circulation. After 24 hours, the patient regained consciousness, and the urine output was 15–30 ml/hour. Also, the blood lactate concentration and heart rate were decreased. Dopamine and epinephrine were continued (8 µg/kg/minute dopamine and 0.08 µg/kg/minute epinephrine). Intravenous methylprednisolone (160 mg/day) was used for the first 3 days, followed by a dose of 80 mg/day, and intravenous immunoglobulin therapy at 25 g/day for 5 consecutive days. In addition, the patient developed severe pneumoniae caused by *Klebsiella pneumoniae*. We used the antibiotics based on the drug susceptibility testing. NT-proBNP, CK-MB, and troponin T levels were decreasing gradually. Echocardiograms showed the LVEF improved gradually (Figs. 1, 2). The patient regained consciousness, command action, and sinus rhythm. ECMO flow rate was gradually decreased to 1.5 L/minute. Respiratory and circulatory function was clinically stable. The 24-hour urine volume was 2400 ml. After 8 days support, he was successfully weaned from ECMO. At this moment, transthoracic echocardiography revealed an abnormal echo in the right atrium (considering thrombosis or neoplasm), about 19.0 × 7.3 mm, attached to the interatrial septum, while left ventricular ejection fraction is 33%. Therefore, oral anticoagulation warfarin (2.5 mg/day) was used. During the following days, marked clinical and laboratory improvement was observed. Steroids were then gradually tapered. The patient was transferred to the ward on day 14. The further clinical course was uneventful. Finally, the patient was discharged on day 45 without complication, with LVEF of 42% (Fig. 2). Also, he took some medications (methylprednisolone 36 mg daily orally, metoprolol succinate 23.75 mg twice daily orally, pantoloc 40 mg daily orally) according to the doctor’s prescription when discharged from hospital**.** Through follow-up by telephone, we learned that the patient died from an unknown reason 1 month after being discharged from the hospital.

## Discussion

We reported the case of fulminant myocarditis occurring as an immune-related adverse event due to nivolumab, which had been successfully treated with combination of ECMO, methylprednisolone, and intravenous immunoglobulin. To the best of our knowledge, this is the fifth case of fulminant myocarditis induced by immune checkpoint inhibitors adjuvant treated with ECMO.

Use of immune checkpoint inhibitors has revolutionized the management of many cancers and results in durable responses in patients with metastatic disease [1]. But the benefits of immune checkpoint inhibitors can be offset by severe immune-related adverse events. These immune-related adverse events can affect almost any organ. Myocarditis is a rare but fatal complication, which can result in refractory cardiogenic shock, chronic heart failure, and fatal arrhythmias [4]. The incidence of immune checkpoint inhibitors-associated myocarditis ranges from 0.1% to 1%. Patients with immune checkpoint inhibitor-associated myocarditis often have a fulminant course with a case fatality rate of 25–50% [5]. Furthermore, fatality rates of myocarditis induced by immune checkpoint inhibitors are as high as 36% with monotherapy and 67% with combination immunotherapy [6].

A systematic review showed that most cases and fatalities of myocarditis occurred shortly after initiation of immune checkpoint inhibitor therapy [7]. The median onset of myocarditis was 27 days (range 5–155 days), with 76% cases occurring in the first 6 weeks of treatment [6]. In the present case, fulminant myocarditis developed after treatment with nivolumab for 4 months. ECMO is a rescue therapy used to stabilize patients with hemodynamic compromise such as refractory cardiogenic shock or cardiac arrest. According to PubMed database search, there are only four case reports on myocarditis caused by immune checkpoint inhibitors nivolumab, pembrolizumab, ipilimumab, or any combination of these agents, adjuvant treated with ECMO (Table 1) [8–11]. This case shows that ECMO is effective as a rescue therapy, which may play an important role in the adjuvant treatment of fulminant myocarditis-induced by immune checkpoint inhibitors.

**Table 1:** Summary of cases with ECMO of myocarditis from immune checkpoint inhibitors.

Authors, year	Age, sex	Malignancy	ICI	Doses prior to myocarditis	Treatments utilized	Outcome
Arangalage *et al*. (2017)	35, F	Melanoma	Ipi 3 mg/kg, Nivo 1 mg/kg	1	Intravenous solumedrol 1 g/day and IVIG followed by ECMO and plasma exchange followed by tacrolimus	Survived
Frigeri *et al*. (2018)	76, F	Metastatic lung adenocarcinoma	Nivo (dose NA)	7	ECMO, IABP, intravenous methylprednisolone 5 mg/kg/day, plasmapheresis, infliximab 5 mg/kg, three doses	Survived
Yamaguchi *et al*. (2018)	60, M	Melanoma	Nivo 2 mg/kg	13	ECMO, IABP, intravenous prednisolone 1000 mg/day for 3 days + IVIG at 50 g/day for 2 days	Survived
Imai *et al*. (2018)	70, M	Squamous cell carcinoma of lung	Pembro (200 mg)	2	Intravenous methylprednisolone (1 g/day) for 3 days, IVIG 1 g/kg for 2 days, ECMO, IABP	Died

*ECMO* extracorporeal membrane oxygenation, *ICI* immune checkpoint inhibitors, *IABP* intraaortic balloon pump, *IVIG* intravenous immunoglobulin, *ipi* ipilimumab, *nivo* nivolumab, *pembro* pembrolizumab, *mg/kg* milligrams/kilogram body weight, *NA* not available

The pathophysiology of myocarditis caused by immune checkpoint inhibitors is still unclear. It has been highlighted that there is a shared antigen or high frequency of T-cell receptor sequences among myocardium, skeletal muscle, and tumors. The mechanism of cardiotoxicity is likely related to the role of PD-1 in cardiomyocyte protection against autoimmune attacks [12]. In the tumor microenvironment, tumor cells commonly express high levels of PD-L1, keeping immune responses in check by preventing CD^8+^ T-cell-mediated killing of cancer cells. PD-1, expressed on the surface of T cells, binds to PD-L1, then inhibiting checkpoint signaling and decreasing T-cell cytotoxicity. Nivolumab is a monoclonal antibody that binds PD-1 and blocks PD-1/PD-L1 interaction, enhancing T-cell cytotoxicity, and increasing cytokine production, ultimately suppressing tumor activity [13]. Because of its clinical benefits, nivolumab is widely used in the treatment of various malignancies.

## Conclusions

In conclusion, fatal myocarditis may develop as an adverse event from the use of immune checkpoint inhibitors. Early recognition and prompt treatment are crucial for improving clinical outcomes. In this case, we suggest that ECMO should be considered in the management of fulminant myocarditis, the severe immune-related adverse effect associated with immune checkpoint inhibitors.

## Data Availability

Data sharing is not applicable to this article as no datasets were generated or analyzed during the current study.

## Abbreviations

ECMO: Extracorporeal membrane oxygenation
CTLA-4: Cytotoxic T-lymphocyte activator-4
PD-1: Programmed-death-1
PD-L1: Ligand programmed death ligand 1
LVEF: Left ventricular ejection fraction
